# Spray-Drying Microencapsulation of Andean Blueberry (*Vaccinium meridionale* Sw.) Anthocyanins Using Prickly Pear (*Opuntia ficus indica* L.) Peel Mucilage or Gum Arabic: A Comparative Study

**DOI:** 10.3390/foods12091811

**Published:** 2023-04-27

**Authors:** Maria Carolina Otálora, Andrea Wilches-Torres, Jovanny A. Gómez Castaño

**Affiliations:** 1 Grupo de Investigación en Ciencias Básicas (NÚCLEO), Facultad de Ciencias e Ingeniería, Universidad de Boyacá, Tunja 150003, Colombia; andreawilches@uniboyaca.edu.co; 2 Grupo Química-Física Molecular y Modelamiento Computacional (QUIMOL^®^), Escuela de Ciencias Químicas, Universidad Pedagógica y Tecnológica de Colombia, Tunja 150003, Colombia

**Keywords:** Andean blueberry, *Opuntia ficus-indica* mucilage, recovery of byproducts, spray-drying microencapsulation, natural dye, anthocyanins

## Abstract

The recovery of byproducts from the food industry is a promising approach to obtain hydrophilic biopolymers with potential health benefits. In this work, the mucilage obtained from the peel of the *Opuntia ficus-indica* (OFI) fruit was compared with gum arabic (GA) as wall materials for the microencapsulation of Colombian blueberry anthocyanins, using the spray-drying process. For both types of microencapsulates, the following were determined: anthocyanin content (UV–vis and HPLC/MS-MS), total dietary content (enzymatic–gravimetric method), antioxidant activity (ORAC), color (CIEL*ab* parameters), morphology (SEM and particle size), and thermal behavior (DSC/TGA). Six different anthocyanins were identified by HPLC/MS-MS in the non-lyophilized Andean blueberry sample (LABP) and in the OFI-mucilage and GA microcapsules. OFI mucilage, compared to GA, favors the formation of larger spherical particles, a smoother surface without cracks, and greater thermal stability. The higher anthocyanin retention capacity in OFI microcapsules leads to higher antioxidant capacity and red coloration for this biomaterial. Consequently, the microencapsulation of anthocyanins with mucilage from the peel of the OFI fruit is proposed as a promising alternative for the protection and incorporation of this natural dye with high antioxidant capacity and dietary fiber content in new functional food/cosmetic formulations, while giving added value to the natural byproducts of OFI.

## 1. Introduction

The incorporation of natural pigments in the formulation of new products in the food, cosmetics, and pharmaceutical industries has become a research area of intense activity and high development. The main objective of this activity is to replace traditional synthetic dyes—such as coal tar derivatives, to which toxic effects are attributed due to the presence of azo groups [1]—with pigments extracted from natural products with desirable nutritional and health benefits for the consumer. Nevertheless, the high proneness of natural pigment molecules to breaking down due to environmental factors (such as water, light, heat, oxygen, relative humidity, etc.) has made this task a challenge for developers of new food formulations. To address this difficulty, the food industry has adopted spray-drying microencapsulation using biopolymers as wall materials as a leading technique to increase the stability and bioaccessibility of sensitive natural pigments [2].

The fruit of the species *Vaccinium meridionale* Swartz is a wild berry (Andean blueberry) found in the mountains of Colombia, Venezuela, and Ecuador, where it is known as “agraz”. This berry contains high concentrations of anthocyanins (flavonoids)—that is, water-soluble glycosylated anthocyanidins—and is of interest in the food industry due to its desired coloring capacity, as well as for its nutritional and medicinal properties [3,4]. Depending on the pH, anthocyanidins can vary from red to black through purple and blue [4]. These compounds have certain anticancer, antidiabetic, and anti-inflammatory properties, due to their high antioxidant capacity [5]. However, anthocyanins are prone to degradation due to pH, temperature, light, oxygen, interaction with metal ions, and association with other compounds (co-pigments, sugars, proteins, degradation products, etc.) [6]. The hydroxylation state of the B ring of the anthocyanidin moiety, along with pH, has been shown to mediate the degradation of anthocyanin to its corresponding phenolic acid and aldehyde [7].

Several studies have shown that microencapsulation by spray-drying favors the preservation and bioaccessibility of anthocyanins extracted from blueberries (*Vaccinium* spp.) [6]. Selected wall materials for anthocyanin microencapsulation include mesquite gum [8], whey protein isolate and gum arabic [9], whey protein isolate [10], maltodextrin DE-20 and hi-maize [11], protein concentrate [12], and maltodextrin and high-amylose-resistant corn starch [11]. However, these encapsulating materials are based on proteins and polysaccharides that do not stand out for providing significant added functional value to the final product, and which present sustainability problems.

On the other hand, the mucilage extracted from cactaceous plants has shown outstanding ability as a functional encapsulant biomaterial because of its gelling and thickening properties, and its high content of dietary fiber [13]. Mucilage is a natural water-soluble biopolymer that is rich in oligosaccharides of an anionic, polyelectronic nature. This polysaccharide biomaterial can be obtained from byproducts (pomace, seeds, peels, stems, etc.) derived from the processing of fresh fruits and plants of the Cactaceae family [14,15]. We have recently shown that the peel of the fruits of the Cactaceae family is a rich source of bioactive compounds (e.g., pigments and polyphenols) with antioxidant activity, as well as dietary fiber, making this plant material a highly underutilized functional ingredient with the potential to be applied in new formulations in the food industry [15]. Likewise, Gheribi, Habibi, and Khwaldia [16] demonstrated that mucilage from *Opuntia ficus-indica* fruit peels can be used as a raw biopolymer material for the development of biodegradable packaging.

This study investigated the applicability of the mucilage of the peels of the *Opuntia ficus-indica* fruit as a wall material for the spray-drying microencapsulation of anthocyanins from the Colombian Andean blueberry. For comparative purposes, the same anthocyanin microencapsulation procedure was also carried out using gum arabic as the traditional wall material. For both microcapsule formulations, the microstructural (SEM and particle size) and thermal (DSC/TGA) properties were determined, as well as the relative percentages and identification of anthocyanins, color parameters, dietary fiber, and antioxidant activity. For some studies, a comparison with the lyophilized fruit pulp without microencapsulation was also performed.

## 2. Materials and Methods

### 2.1. Chemical and Reagents

The solvents acetonitrile (41.05 g/mol, ≥99.9%, CAS No 75-05-8) and formic acid (46.03 g/mol, >98%, CAS No 64-18-6) were purchased from Merck (Darmstadt, Germany). 2,2′-Azobis(2-amidinopropane) (AAPH), 6-hydroxy-2,5,7,8-tetramethylchroman-2-carboxylic acid (Trolox), and cyanidin-3-glucoside were purchased from Sigma-Aldrich (St. Louis, MO, USA).

### 2.2. Vegetal Materials

Fresh Andean blueberry (*Vaccinium meridionale* Sw.) fruits were collected from wild shrubs in the vicinity of the municipality of Raquira, located at 2150 m above sea level (5°32′20″ N; 73°37′48″ O) and with an average temperature of 16 °C, in the department of Boyacá, Colombia. The black–violet fruits were selected, washed, chopped, and then homogenized in a blender at minimum power for 1 min. The resulting product was frozen at −80 °C in an ultralow-temperature freezer (Buzzer, model MDF-86V188E, Shanghai, China) for 48 h. The samples were then lyophilized in a 4.5 L FreeZone lyophilizer (Labconco, Kansas City, MO, USA) at −84 °C under a 0.13 mbar vacuum for 48 h. After lyophilization, the samples were ground in a food processor and stored at a refrigerated temperature (4 °C) in amber bottles until further analysis. Non-microencapsulated lyophilized Andean berry pulp (LABP) was designated as the control sample.

Mucilage was extracted from *Opuntia ficus-indica* fruit peels collected from local food restaurants, following the method described by Otálora et al. [14]. Prickly fresh peels were washed with distilled water at room temperature and cut into small pieces. The small pieces of the prickly fruit peels were placed into 100 mL beakers, to which distilled water was added at room temperature at a 1:2 *w/v* ratio (peel:water), and left for 12 h. The hydrated peels were manually squeezed to extract their gel. Then, 95% ethanol at 18 °C was added to the extracted gel at a 3:1 ratio (ethanol:gel), and the mixture was allowed to stand for 15 min without stirring until the formation of a milky-white supernatant corresponding to the fruit peel mucilage. The mucilage was collected and then dried in an oven at 50 °C for 3 h. The dry material was manually macerated in a porcelain mortar and, subsequently, filtered through a 60-mesh sieve until a fine powder was obtained (standard granulometry ≤ 250 µm). The powdered mucilage was placed in high-density polyethylene bags and stored in a desiccator, at room temperature, with a relative humidity of 30%, until characterization.

### 2.3. Spray-Drying Microencapsulation

Two different microcapsule formulations were prepared according to the design shown in Table 1. The LABP:wall material ratios in the formulations of the feed mixes were determined on the basis of the literature [17], where the range of total solids content was below 6%, suggested as the most suitable for spray-drying when only mucilage is used as the wall material. Next 1.0 g of mucilage obtained from the peels of the prickly pear and 1.2 g of gum arabic (conventional microcapsule wall material of high cost and restricted supply [18]) were dissolved separately in 100 mL of distilled water at 18 °C. To ensure complete solubilization, both solutions were constantly stirred at 300 rpm for 6 h and 30 min, respectively, using a magnetic stirrer (C-MAG HS 7 S000, IKA, Staufen im Breisgau, Germany). Then, 6.0 g of LABP was mixed with 100 mL of each of the two prepared solutions. In each case, the feed mixture was kept under constant magnetic stirring at room temperature until homogeneous. The total solid content of the feed mixtures was 6.0% for the SD-MP microcapsules (i.e., LABP/mucilage obtained from prickly pear rind) and 6.2% for the SD-GA microcapsules (i.e., LABP/gum arabic). Each mixture was then fed into a mini spray-dryer (B-290, Büchi Labortechnik, Switzerland) with the suction rate maintained at 86% to maximize the cyclone separation rate, and a compressed air pressure of 40 bar. The spray-dryer used a nozzle with an internal diameter of 0.7 mm, a feed flow of 350 mL/h, and an inlet air temperature of 170 °C. The two microencapsulates that were obtained—i.e., SD-MP and SD-GA—were stored in the dark at room temperature for further analysis and use.

**Figure 1:**
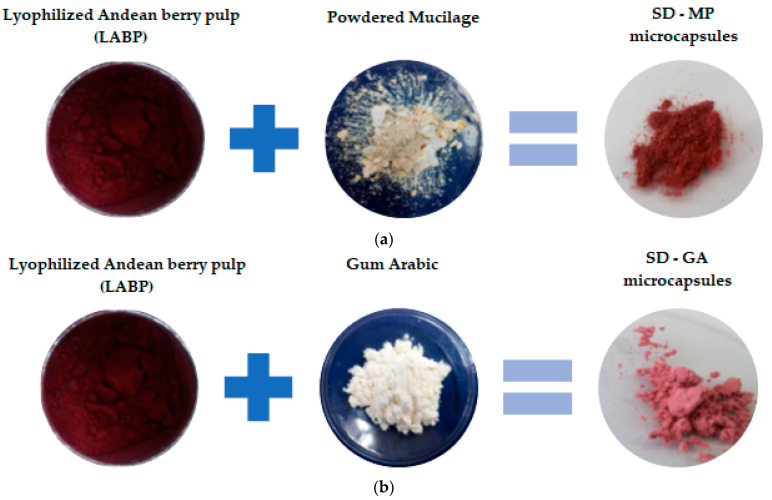
Selected photographs of the Andean berry pulp microencapsulation process using prickly pear fruit peel mucilage (**a**) and gum arabic (**b**) as the encapsulating materials.

Figure 1 shows photographs of the main stages involved in the Andean berry pulp microencapsulation process using prickly pear fruit peel mucilage and gum arabic. The process yield (PY) was determined as the ratio of the dry weight of the powder (g) after the spray-drying process to the initial amount of solids in the feed solution (g) [19].

### 2.4. Physicochemical and Functional Characterization of Microcapsules

#### 2.4.1. Total Anthocyanin Contents

The total anthocyanin contents in the microcapsules, obtained on the following day after drying and storage in the dark at room temperature, were determined using the method reported by Garzón et al. [20], with some modifications. First, 15.67 mg of LABP, 11.27 mg of SD-MP, and 127.53 mg of SD-GA were separately mixed with 50 mL of ethanol and water (acidified with 1% HCl) (80:20), and the solutions were stirred at room temperature and then centrifuged (5000× *g* rpm, 20 min, 4 °C). The absorbance of each solution was measured at 535 nm using a Macherey-Nagel (Düren, Germany) Nanocolor UV/VIS II spectrophotometer. The total anthocyanin (TA) concentration in the extracts was determined and expressed as cyanidin-3-O-glucoside (C-3-O-G) according to Equation (1). All results were expressed as mg C-3-O-G equivalents/L of samples.

(1) $$TA=\left(A\times Mw\times DF\times V\times 10^{3}\right)/\left(m\times \varepsilon \right)$$

where *A* = (absorbance at 535 nm), *M_W_* = the molecular weight of C-3-O-G (449.2 g/mol), *DF* = the dilution factor, *V* = the total volume of the diluted samples (mL), *ε* = the molar extinction coefficient of C-3-O-G (26,900 mol/L × cm), and *m* = the weight of samples (g).

#### 2.4.2. Analysis of Anthocyanins by HPLC–MS

The identification of anthocyanins in the LABP, SD-MP, and SD-GA samples was performed using an Acquity H Class Plus Ultra-High-Performance Liquid Chromatography (UPLC) device coupled a with Xevo-G2-XS Quadrupole Time-of-Flight (QTOF) detector (Waters Corporation, Milford, MA, USA). These UPLC-QTOF analyses were performed in positive electrospray ionization (ESI) mode. For extracting anthocyanin compounds, 35.2; 32.8, and 34.6 mg of LABP, SD-MP, and SD-GA, respectively, were extracted with a methanol:water mixture (70:30, *v*/*v*) acidified with trifluoroacetic acid (1%) under agitation in a vortex for 45 min, centrifuged at 5000× *g* rpm for 20 min at 4 °C, and the supernatant was filtered through a 0.45-micron Millipore filter. Aliquots (5 µL) were separated through a PREMIER BEH C18 AX analytical column (2.1 mm × 50 mm, 1.7 µm particle size) (Waters, Milford, CT, USA). The eluent system was composed of type I water and 0.1% formic acid (solvent A), and acetonitrile and 0.1% acid formic (solvent B), at a flow rate of 0.5 mL/min. The gradient program was 95:5 *v*/*v* at 0 min, 80:20 *v*/*v* at 10 min, 60:40 *v*/*v* at 14 min, and 95:5 *v*/*v* at 15 min. The following parameters were maintained: source temperature of 120 °C, desolvation temperature of 500 °C, desolvation gas flow rate of 900 L/h, and cone gas flow rate of 100 L/h. The cone and capillary voltages were set at 40 V and 2.0 kV, respectively.

#### 2.4.3. Antioxidant Activity by Oxygen Radical Absorbance Capacity (ORAC)

Antioxidant capacity based on oxygen radical absorbance capacity (ORAC) was measured using the method reported by Prior et al. [21]. First, 101.90 mg of LABP, 101.52 mg of SD-MP, and 101.62 mg of SD-GA were dispersed separately in 10 mL of distilled water, and then the mixtures were stirred at room temperature and filtered through a Millipore membrane. Aliquots of 150 μL of fluorescein solution (1 μM), 25 μL of each solution, and 25 μL of AAPH (250 mM) were added to a microplate and incubated for 30 min at 37 °C. The fluorescence signal of each sample was measured at 528 nm (emission) and 485 nm (excitation) at 2 min intervals for 2 h at 37 °C, using a spectrofluorometer microplate reader (BioTek, Synergy HT Microplate Reader, Winooski, VT, USA). For the calibration curve, Trolox solutions of 10, 25, 50, 100, 150, 200, 400, and 500 μM were used. The results were expressed in µmol of Trolox equivalents/100 g of sample.

#### 2.4.4. Dietary Fiber Content

The total dietary fiber (TDF) contents in the powdered mucilage and in the SD-MP and SD-GA microcapsules were determined using a total dietary fiber test kit (TDF-100A) provided by Sigma-Aldrich (St. Louis, MO, USA), based on the enzymatic–gravimetric method AOAC 985.29 [22].

#### 2.4.5. Color Parameters

The color attributes (L*, a*, and b*) of the powdered mucilage and gum arabic, as well as of the SD-MP and SD-GA microcapsules (samples placed in Petri dishes), were determined randomly on three different surfaces using a Chroma Meter CR-300 (Konica Minolta Co., Osaka, Japan), and their intensity was recorded accordingly. The instrument was calibrated with a white tile calibration plate (L* = 94.30; a* = −0.20; b* = 3.70). The results were expressed as the CIE L*, a*, and b* color parameters, where L* represents the lightness, a* represents the redness (+) to greenness (−), and b* represents the yellowness (+) to blueness (−) of the samples.

The derived color parameters of chroma ($C_{ab}^{*}$) and hue (*h_ab_*) were calculated using Equations (2) and (3), respectively [23]:

(2) $$C_{ab}^{*}={\left[{\left(a^{*}\right)}^{2}+{\left(b^{*}\right)}^{2}\right]}^{1/2},$$

(3) $$h_{ab}=arctan\;\left[b^{*}/a^{*}\right],$$

#### 2.4.6. Particle Size and Polydispersity Index

The particle size distribution, average diameter, and polydispersity index of the SD-MP and SD-GA microcapsules were determined by the laser diffraction technique using a NanoPlus zeta/nanoparticle analyzer (Micromeritics Instrument CORP, Norcross, GA, USA). The SD-MP and SD-GA microcapsules were diluted in type I water to adjust the obscuration range as follows: viscosity of 0.8878 cP, average refractive index of 1.33, and sample temperature of 25 °C, in a glass cuvette.

#### 2.4.7. Scanning Electron Microscopy (SEM)

The microscopic morphology of the SD-MP and SD-GA microcapsules was evaluated by scanning electron microscopy (SEM) using an EVO MA 10-Carl Zeiss device (Oberkochen, Germany) operating at 20 kV. All samples were coated using gold–palladium sputtering before their examination.

#### 2.4.8. Thermal Characterization

Thermogravimetric analysis (TGA) and differential scanning calorimetry (DSC) of the gum arabic and the SD-MP and SD-GA microcapsules were performed using a TA Instrument (SDT Q600 V20.9 Build 20, New Castle, DE, USA). Argon was used as a purge gas (100 mL/min). The dried samples were placed in aluminum pans and heated from 20 to 600 °C at a heating rate of 10 °C/min.

### 2.5. Statistical Analysis

The total anthocyanin contents and antioxidant capacity (ORAC), along with the color parameter data presented in Table 1 and Table 2, are reported as the mean ± standard deviation (*n* = 3). Data were analyzed using analysis of variance (ANOVA), and means were compared using Fisher’s least significant difference test (*p* < 0.05).

## 3. Results and Discussion

### 3.1. Antioxidant Capacity and Contents of Anthocyanins and Dietary Fiber

As shown in Table 2, SD-MP microcapsules presented a low yield of the final product, in contrast to the SD-GA microcapsules, possibly due to mucilage—a compound with adhesive properties due to its high sugar content—increasing the tendency for microparticles to adhere to the walls of the drying chamber during the process [19]. A 47% and 90% decrease in anthocyanin content was observed in the SD-MP and SD-GA microcapsules, respectively, compared to the contents originally present in the non-microencapsulated freeze-dried pulp (LABP). This decrease in anthocyanins in the encapsulated samples can be directly attributed to the rate of degradation due to mechanical and thermal effects during the homogenization process with the wall material and the subsequent spray-drying, respectively [24]. However, the fivefold higher concentration of anthocyanins in the SD-MP microcapsules compared to the SD-GA microcapsules reveals a remarkable pigment-protecting ability of OFI peel mucilage over gum arabic, attributable to the high emulsifying capacity of the mucilage, which contributes to the greater physical stability of the feed mixture during the spray-drying process [25]. The low content of anthocyanins in the SD-GA microcapsules may have been due to the high branching and high degree of polymerization of the gum arabic molecules, which would cause a low retention of the natural pigment during microencapsulation by spray-drying [26].

As expected, the higher retention of anthocyanins in the SD-MP microcapsules gave them a higher antioxidant activity than the SD-GA microcapsules (Table 2). In fact, the SD-MP sample revealed an even higher antioxidant activity than the non-microencapsulated sample (LABP); this was attributed to the contents of phenolic compounds (25.0 g of GAE/100 g of sample) in the mucilage used as the wall material in the formulation of SD-MP microcapsules [14]. This result is consistent with the findings of Tabio-García et al. [27], who determined that the addition of *Opuntia ficus-indica* cactus mucilage improved the antioxidant activity of amaranth microcapsules.

As also shown in Table 2, both kinds of microcapsules had significant contents of total dietary fiber, with a higher contribution for gum arabic microcapsules (63%) compared to OFI mucilage microcapsules (35%). This result is consistent with the high dietary fiber content reported for gum arabic used in food mixes; for example, an 80% TDF content in GA was reported by Mariod [28], while values close to 56% of dietary fiber have been reported for OFI mucilage. For example, Otálora et al. [17] determined that the addition of mucilage from *Opuntia ficus-indica* cladodes improved the total dietary fiber content of pink guava microcapsules. Therefore, both types of microcapsules could be applied as additives in the development of nutritionally enhanced food products, since their incorporation—for example, in dairy products—could reduce the fat content by adding them in powder form.

### 3.2. CIELab Color Space

As shown in Table 3, the microcapsules formulated with 1.2% gum arabic (i.e., SD-GA) presented a lightness value significantly higher (∆L* = +10.7) than that recorded for the microcapsules formulated with 1.0% OFI mucilage (i.e., SD-MP), which was attributed to the whiter color and higher contents of the gum arabic powder compared to the color and contents of the mucilage powder [29]. The range of values of the parameter a* (15 ≤ a* ≤ 25) indicated that the color of both microcapsules was framed in red tones, which is consistent with the presence of anthocyanins in the obtained microcapsules [8]. The highest value of the parameter a* for SD-GA microcapsules (i.e., a dark red) is consistent with the highest retention of anthocyanins [30], as indicated in Table 2. A similar result was reported by de Araujo et al. [31] for pomegranate powder using a mixture of gum arabic and capsules as wall materials. On the other hand, the value of the parameter b* was higher for the SD-MP microcapsules (∆b* = +3.6), which coincides with the yellower hue of the OFI mucilage compared to the hue of the gum arabic. Similar results were reported by Ahmada et al. [32] during the spray-drying microencapsulation of saffron anthocyanins using β-glucan and β-cyclodextrin as wall materials.

The chroma parameter ($C_{ab}^{*}$), which is related to the purity of the particles, was lower for the SD-MP microcapsules compared to the SD-GA microcapsules (Table 3); that is, the SD-MP microcapsules presented a chroma value close to zero, which is consistent with a higher concentration of the natural pigment in this sample [33]. Finally, the hue (*h_ab_*) of the particles coincided with a pure red hue in the color space, which was lower for SD-GA compared to SD-MP microcapsules. It was observed that the original hue of the gum arabic (L* = 72.21 ± 0.02, a* = 3.58 ± 0.01, and b* = 9.28 ± 0.04) and of the mucilage (L* = 46.84 ± 0.02, a* = 9.15 ± 0.02, and b* = 19.67 ± 0.02)—located in the range from white to yellow—managed to influence the tonality of the encapsulations. Bhagya Raj and Dash [34] reported a similar behavior during the microencapsulation of betacyanins from dragon fruit rind using gum arabic/sodium alginate and gelatin/sodium alginate by spray-drying.

### 3.3. Anthocyanin Identification by HPLC-MS/MS

The HPLC-MS/MS analysis of the LABP, SD-MP, and SD-GA samples of our Andean blueberries led to the identification of six anthocyanin molecules, whose structural details and relative percentages are shown in Table 4.

In all cases, the two cyanidin-derived anthocyanins (i.e., cyanidin-3-O-galactoside (ideain) and cyanidin-3-O-alpha-arabinopyranoside) constituted the two dominant pigments by far (83.3 ≤ anthocyanin % ≤ 99.6) in the samples. Although the anthocyanin ideain had a slightly higher concentration than cyanidin-3-O-alpha-arabinopyranoside in the unencapsulated sample (LABP), in the SD-MP and SD-GA microcapsules the ideain concentration was reduced compared to its cyanidin analog, indicating a greater thermal susceptibility of the former compared to the latter during the spray-drying process. The anthocyanins peonidin-3-O-alpha-arabinoside, delphinidin-3-arabinoside, and delphinidin-3-pyranoside presented lower but significant concentrations (3.5 ≤ anthocyanin % ≤ 7.4) in the LABP sample; however, their concentrations in both microcapsule samples (SD-MP and SD-GA) were found to be below 1%, possibly indicating greater thermal sensitivity of these natural dyes during the spray-drying process. For its part, the anthocyanin with the highest molecular weight (i.e., delphinidin-3-(6-p-coumaroyl-glucoside)) maintained its concentration (0.1%) in all samples.

The HPLC chromatograms and mass spectra of the two most abundant anthocyanins (i.e., ideain and cyanidin-3-O-alpha-arabinopyranoside) found in the Andean blueberry samples LABP, SD-MP, and SD-GA are presented in the Supplementary Materials as Figures S1–S5.

### 3.4. Microscopic Morphology and Particle Size

**Figure 2:**
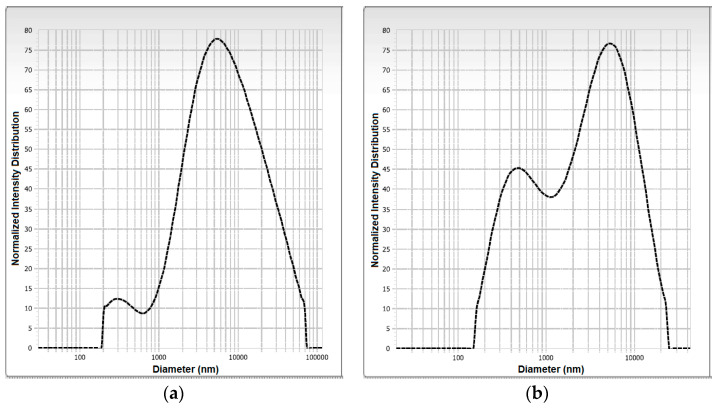
Particle size distribution plots of SD-MP (**a**) and SD-GA (**b**) microcapsules.

The distribution of the particle size and the average diameter of the SD-MP and SD-GA microcapsules are shown in Figure 2. The distribution of the particle size for both types of microcapsule showed a behavior of the bimodal type, indicating a degree of heterogeneity in the particle size; this behavior can influence the powder properties (i.e., appearance, dispensability, and flowability) and the dissolution of the powder when applied to foods, which can affect the texture and sensory characteristics of the food matrix [35]. A bimodal particle size distribution was observed in microcapsules of gallic acid produced with aloe vera (*Aloe barbadensis* Miller) mucilage as a wall material [36]. The SD-MP microcapsules presented an average particle diameter of 11.83 ± 1.52 µm, while the SD-GA microcapsules presented an average particle diameter of 3.16 ± 0.29 µm. These values indicate that the obtained powders could be considered microcapsules [37], with a desirable size (i.e., <100 μm) that would not affect the sensory characteristics when applied in a food matrix [38]. This result is consistent with the relatively large particle sizes (15 ≤ Ø ≤ 27 µm) that we have recently reported for microcapsules of mucilage extracted from cacti [28], attributable to the high gelatinization and crosslinking capacity—as well as the high molecular weight [32,39]—of these polysaccharide-based biomaterials. C. de Campo et al. [40] also observed that the presence of cladode mucilage from the *Opuntia monacantha* cactus in the zeaxanthin nanoparticle formulation increased the particle size. Finally, the increase in the particle size of the SD-MP powder was consistent with the presence of anthocyanins and was correlated with thermal stability of the microcapsules (see Section 3.5), in contrast to the SD-GA powder.

The polydispersity index of the SD-MP microcapsules (1.04) was higher than that of the SD-MD microcapsules (0.54), indicating a fairly narrow size distribution of particles in the latter, suggesting a good solubility (span value < 2) when they are incorporated into a food matrix as a coloring additive, because they will not easily form agglomerates [41].

**Figure 3:**
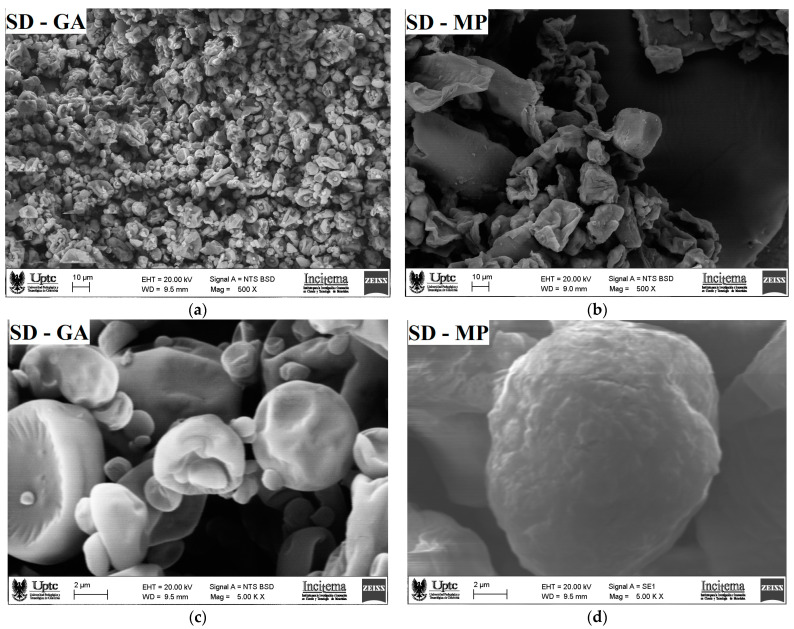
SEM micrograph images taken at 500× (**a**,**b**) and at 5000× (**c**,**d**) of SD-GA and SD-MP microcapsules, respectively.

SEM micrographs of the SD-MP and SD-GA microcapsules taken at 500× and 5000× magnifications are shown in Figure 3. As shown in Figure 3a, the surface structure of the SD-GA microcapsules, viewed at 500× magnification, shows a clumping (sticking) effect between the particles. This attraction effect among the gum arabic microcapsules can be attributed to electrostatic and van der Waals forces characteristic of samples with high amounts of carbohydrates [38]. A similar morphological structure was reported by Mahdi et al. [42] in citron extract microcapsules using gum arabic as an encapsulating agent. In contrast, SD-MP microcapsules (Figure 3b) observed at the same magnification showed a lower degree of agglomeration, attributed to the larger particle size, leading to fewer particle–particle interactions [36]. As for the SEM micrographs taken at 5000× magnification, the SD-GA microcapsules presented irregular and heterogeneous particle shapes, many of which had dented and rough surfaces (Figure 3c), possibly originating from the effects of the particle contraction, followed by incomplete expansion during the spray-drying process [43]. Adsare and Annapure [44] found similar morphological characteristics in curcumin microcapsules using gum arabic as a wall material. Meanwhile, SD-MP microcapsules observed at 5000× magnification (Figure 3d) revealed a more spherical shape—uniform in appearance, with smoother surfaces and fewer cracks. This more homogeneous morphology for the mucilage microcapsules is consistent with its polydispersity index (span value of 1.04) and larger particle size (11.83 ± 1.52 µm), which contribute to a more efficient and stable loading of the pigment. C. de Campo et al. reported similar morphological characteristics [40] for zeaxanthin nanocapsules using *Opuntia monacantha* cactus cladode mucilage as a wall material.

### 3.5. Thermal Characterization

**Figure 4:**
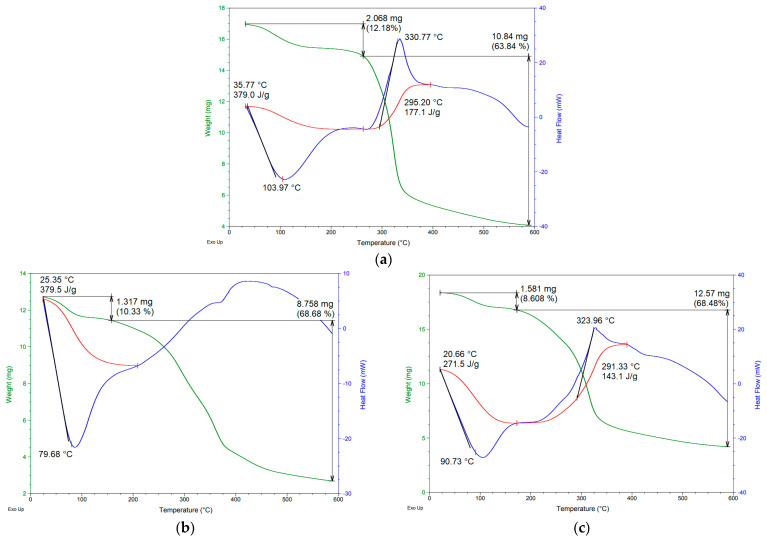
TGA/DSC thermograms of gum arabic (**a**) and of SD-MP (**b**) and SD-GA (**c**) microcapsules. Heat capacity (J/g·°C) (red line).

The thermal behaviors of gum arabic and the SD-GA and SD-MP microcapsules are shown in Figure 4. The thermograms of the wall materials show the typical thermal characteristics reported for this mucilage [14] and for gum arabic [44] (Figure 4a).

The thermogram of the SD-MP microcapsules (Figure 4b) revealed two main thermal events: Firstly, an endothermic event occurred between 25 and 175 °C, with a related mass loss of 10.33%. This event was associated with the loss of free water and water evaporation in the powders. This behavior may have been due to the hydrophilic nature of the functional groups of the polysaccharides present in the mucilage wall material, which allowed the powder to absorb water after the drying process, causing a decrease in Tg (79.68 °C) in contrast to the Tg (90.73 °C) of the SD-GA microcapsules; that is, SD-MP powders should be stored at temperatures below 80 °C to avoid structural changes, such as agglomeration into hard pieces or even solidification of all powder [45]. Likewise, the Tg value represents the interaction and crosslinking density between the components of the Andean berry pulp and the structure of the polymeric chain of the mucilage [28]. The second event corresponded to an exothermic process that occurred between 175 and 400 °C (peak around 375 °C), with a mass loss of 68.68%. This event was attributed to the degradation and volatilization of the polysaccharides that make up the structure of the mucilage [16]. Similar thermal behaviors were observed in thin zeaxan nanoparticles using *Opuntia monacantha* cactus cladode mucilage as a wall material [40], as well as in amaranth extract microcapsules using *Opuntia ficus-indica* cactus cladode mucilage as an encapsulating agent [27].

The thermogram of the SD-GA microcapsules (Figure 4c) showed an initial endothermic event that occurred between 25 and 175 °C, with a weight loss of 8.60%. This was attributed to the evaporation of water that was adsorbed and structurally incorporated into the material. The Tg of 90.73 °C could be attributed to a binary mixture of Andean berry pulp and gum arabic, with a structural interaction between these two structures [44]. The second thermal event was exothermic and occurred between 175 and 400 °C (with a peak of 323.96 °C), with a mass loss of 68.48%. This was attributed to decomposition/volatilization of the microcapsule material. Similar thermal behavior was observed in curcumin microcapsules [44] and finger citron extract microcapsules [42], using gum arabic as the wall material in both cases.

The microcapsules containing mucilage had a significant mass loss around 375 °C in contrast to the microcapsules made up of gum arabic, which had their greatest mass loss at approximately 323.96 °C. This result shows that the SD-MP microcapsule powder has superior thermal stability, possibly due to the higher molecular weight of the hydrocolloid [41]. Therefore, SD-MP microcapsules can be used as a coloring additive in the food industry.

## 4. Conclusions

In this work, a comparative analysis of the properties of two different microcapsules made by spray-drying of Andean blueberry anthocyanins was carried out. One of the microencapsulates used the mucilage extracted from the peels (byproducts) of the prickly pear *Opuntia ficus-indica* as an alternative encapsulation material, while the other microencapsulate used gum arabic as a traditional encapsulation material.

The microcapsules obtained with OFI mucilage (SD-MP) presented an anthocyanin loading capacity approximately five times higher than that of the microcapsules formed using gum arabic (SD-GA). This difference was associated with the formation of larger spherical microcapsules with a smoother surface and with greater thermal resistance when OFI mucilage was used as structural material. This result was attributed to the greater emulsifying and gelling properties of OFI mucilage compared to gum arabic, allowing a superior thermal protection and stabilization effect on the anthocyanins contained in the SD-MP microcapsules. Consequently, the mucilage microcapsules presented a higher antioxidant capacity than the unencapsulated Andean blueberry pulp (LABP) and that encapsulated with gum arabic (SD-GA), caused by a contributory effect between the high load of anthocyanins and the presence of polyphenols from the OFI mucilage. As expected, the SD-MP microcapsules were characterized by a greater red hue due to their higher anthocyanin contents. Additionally, the structure and relative percentage of anthocyanins present in both the unencapsulated lyophilized sample (LABP) and the SD-MP and SD-GA microcapsules were elucidated using the HPLC-MS/MS technique. Here, cyanidin-derived anthocyanins (i.e., ideain and cyanidin-3-O-alpha-arabinopyranoside) mostly dominated the anthocyanin contents in all samples. On the other hand, microcapsules of gum arabic (SD-GA) presented higher contents of total dietary fiber.

In summary, it can be seen that the mucilage extracted from OFI prickly pear peels has advantageous properties as an encapsulating material for highly antioxidant pigments, such as Andean blueberry anthocyanins. This condition gives the mucilage microcapsules containing Andean blueberry anthocyanins the potential to be used in the food industry as water-soluble natural colorants for the formulation of novel and functional products (antioxidant + dietary fiber) that can be classified with the “clean label”; however, it is necessary to take into account different aspects that should be deeply investigated in the future, including evaluation of the behavior of the microencapsulates as coloring additives in the processing of model food systems, and their sensory acceptance by consumers, as well as the effects of the microencapsulation on the bioaccessibility and bioavailability of bioactive compounds.

## 5. Patents

The results of this work are a structural part of the National Invention Patent Application No. NC2022/0007738, submitted for evaluation to the Superintendencia de Industria y Comercio of Colombia.

In compliance with the provisions of the Contracts for Access to Genetic Resources and its derivative products No. 364 and 365 of 2022, signed between the Ministerio de Ambiente y Desarrollo Sostenible of Colombia and the Universidad de Boyacá and the Universidad Pedagógica y Tecnológica de Colombia, respectively, it should be clarified that the genetic material (that is, the mucilage samples) was extracted from *Opuntia ficus-indica* cactus fruits of Colombian origin.

## Figures and Tables

**Table 1:** Proportions of LABP and wall materials in the microcapsule formulations.

Microcapsules	Proportion (LABP (g): Wall Material (mL)) [g LABP:g Wall Material]
SD-MP	(6.0:100) [6.0:1.0]
SD-GA	(6.0:100) [6.0:1.2]

**Table 2:** Process yield (PY), total anthocyanin content (TAC), antioxidant capacity (ORAC), and dietary fiber content of SD-MP and SD-GA microcapsules, together with the respective LABP values.

Parameter	LABP	SD-MP	SD-GA
PY (%)		23.90	69.22
TAC ^1^	2057.54	1082.08	216.13
ORAC ^2^	97.95 ± 0.54 ^b^	100.05 ± 0.07 ^a^	84.31 ± 4.00 ^c^
TDF ^3^	-	34.81	63.24

^1^ TAC is represented as mg of C-3-O-G equivalents/L of sample. ^2^ ORAC is represented as µmol of Trolox equivalents/g of sample in dry. ^3^ TDF is expressed as g/100 g. Different letters in the same row and column for each parameter indicate a statistical difference (*p* < 0.05) between samples.

**Table 3:** CIEL*ab* color parameters determined for spray-dried microcapsules of gum arabic (SD-GA) and OFI mucilage (SD-MP) containing Andean blueberry anthocyanins.

Color Parameter	SD-MP	SD-GA
L* (luminosity)	30.78 ± 0.49 ^b^	41.52 ± 0.05 ^a^
a*	18.13 ± 0.14 ^b^	24.18 ± 0.00 ^a^
b*	8.27 ± 0.01 ^a^	4.70 ± 0.00 ^b^
$C_{ab}^{*}$ (chroma)	19.93 ± 0.13 ^b^	24.64 ± 0.01 ^a^
h_ab*_ (hue)	24.51 ± 0.16 ^a^	11.02 ± 0.02 ^b^

Different letters in the same row for each parameter indicate a statistical difference (*p* < 0.05) between samples.

**Table 4:** Identities and percentages of the anthocyanins present in unencapsulated Andean blueberry samples (LABP) and spray-dried samples microencapsulated with OFI mucilage (SD-MP) and gum arabic (SD-GA), as determined by HPLC-MS/MS.

Basic Structure	Name	Formula	R^3^	R^5^	R^6^	R^7^	R^3′^	R^4′^	R^5′^	[M + H]^+^	LABP	SD-MP	SD-GA
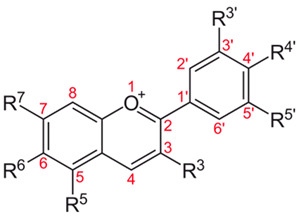	Peonidin-3-O-alpha-arabinoside	C_21_H_20_O_10_	O-α-Ara	OH	H	=O	H	OH	OMe	433.11	3.5%	0.1%	0.1%
	Delphinidin-3-arabinoside	C_20_H_19_O_11_	O-α-Ara	OH	H	OH	OH	OH	OH	435.09	7.4%	0.5%	0.1%
	**Cyanidin-3-O-alpha-arabinopyranoside**	**C_20_H_19_O_10_**	**O-Arapyr**	**OH**	**H**	**OH**	**H**	**OH**	**H**	**419.09**	**40.4%**	**60.0%**	**52.7%**
	Delphinidin-3-pyranoside	C_21_H_21_O_12_	O-Pyr	OH	H	OH	OH	OH	OH	465.10	5.6%	0.5%	0.1%
	**Cyanidin-3-O-galactoside (Ideain)**	**C_21_H_21_O_11_**	**O-Gal**	**OH**	**H**	**OH**	**OH**	**OH**	**H**	**449,11**	**42.9%**	**38.8%**	**46.9%**
	Delphinidin-3-(6-*p*-coumaroyl-glucoside)	C_30_H_27_O_14_	O-6p-Cou	OH	H	OH	OH	OH	OH	611,14	0.1%	0.1%	0.1%

## Data Availability

The data presented in this study are available upon request from the corresponding author.

## Supplementary Materials

The following supporting information can be downloaded at: https://www.mdpi.com/article/10.3390/foods12091811/s1, Figure S1: HPLC chromatograms of the two main anthocyanins (i.e., cyanidin-3-O-alpha-arabinopyranoside (C_20_H_19_O_10_) (top) and cyanidin-3-O-galactoside (C_21_H_21_O_11_) (bottom)) identified in the pulp of Colombian Andean blueberry (*Vaccinium meridionale Sw*). Figure S2: Mass spectrum of the anthocyanin cyanidin-3-O-alpha-arabinopyranoside (C_20_H_19_O_10_) identified in the pulp of Colombian Andean blueberry (*Vaccinium meridionale Sw*), determined using the HPLC/MS-MS technique. Figure S3: Mass spectrum of the anthocyanin cyanidin-3-O-galactoside (C_21_H_21_O_11_) identified in the pulp of Colombian Andean blueberry (*Vaccinium meridionale Sw*), determined using the HPLC/MS-MS technique. Figure S4: HPLC chromatograms of the two main anthocyanins (i.e., cyanidin-3-O-alpha-arabinopyranoside (C_20_H_19_O_10_) (top) and cyanidin-3-O-galactoside (C_21_H_21_O_11_) (bottom)) identified in the Andean blueberry (*Vaccinium meridionale Sw*) microcapsules, using OFI mucilage as the encapsulation material. Figure S5: HPLC chromatograms of the two main anthocyanins (i.e., cyanidin-3-O-alpha-arabinopyranoside (C_20_H_19_O_10_) (top) and cyanidin-3-O-galactoside (C_21_H_21_O_11_) (bottom)) identified in the Andean blueberry (*Vaccinium meridionale Sw*) microcapsules, using gum arabic as the encapsulation material.

## References

[1] Dey S., Nagababu B.H. (2022). Applications of food color and bio-preservatives in the food and its effect on the human health. Food Chem. Advan..

[2] Niaz K., Khan F., Silva A.S., Nabavi S.F., Nabavi S.M. (2020). Analysis of polyphenolics. Recent Advances in Natural Products Analysis.

[3] Garzon G.A., Soto C.Y., Lopez-R M., Riedl K.M., Browmiller C.R., Howard L. (2020). Phenolic profile, in vitro antimicrobial activity and antioxidant capacity of Vaccinium meridionale Swartz pomace. Heliyon.

[4] Agudelo C.D., Luzardo-Ocampo I., Campos-Vega R., Loarca-Piña G., Maldonado- Celis M.E. (2018). Bioaccessibility during in vitro digestion and antiproliferative effect of bioactive compounds from Andean Berry (*Vaccinium meridionale* Swartz) juice. J. Agric. Food Chem..

[5] Shahidi F., Ambigaipalan P. (2015). Phenolics and polyphenolics in foods, beverages and spices: Antioxidant activity and health effects–A review. J. Funct. Foods.

[6] Herrera-Balandrano D.D., Chai Z., Beta T., Feng J., Huang W. (2021). BlueBerry anthocyanins: An updated review on approaches to enhancing their bioavailability. Trends Food Sci. Technol..

[7] Woodward G., Kroon P., Cassidy A., Kay C. (2009). Anthocyanin Stability and Recovery: Implications for the Analysis of Clinical and Experimental Samples. J. Agric. Food Chem..

[8] Jiménez-Aguilar D.M., Ortega-Regules A.E., Lozada-Ramírez J.D., Pérez-Pérez M.C.I., Vernon-Carter E.J., Welti-Chanes J. (2011). Color and chemical stability of spray-dried blueBerry extract using mesquite gum as wall material. J. Food Compos. Anal..

[9] Flores F.P., Singh R.K., Kerr W.L., Pegg R.B., Kong F. (2014). Total phenolics content and antioxidant capacities of microencapsulated blueBerry anthocyanins during in vitro digestion. Food Chem..

[10] Flores F.P., Singh R.K., Kerr W.L., Phillips D.R., Kong F. (2015). In vitro release properties of encapsulated blueBerry (*Vaccinium ashei*) extracts. Food Chem..

[11] Rosa J.R., Nunes G.L., Motta M.H., Fortes J.P., Cezimbra Weis G.C., Rychecki Hecktheuer L.H., Muller E.I., de Menezes C.R., da Rosa C.S. (2019). Microencapsulation of anthocyanin compounds extracted from blueBerry (*Vaccinium* spp.) by spray drying: Characterization, stability and simulated gastrointestinal conditions. Food Hydrocoll..

[12] Rocha J.d.C.G., de Barros F.A.R., Perrone Í.T., Viana K.W.C., Tavares G.M., Stephani R., Stringheta P.C. (2019). Microencapsulation by atomization of the mixture of phenolic extracts. Powder Technol..

[13] Ribeiro J.S., Veloso C.M. (2021). Microencapsulation of natural dyes with biopolymers for application in food: A review. Food Hydrocoll..

[14] Otálora M.C., Wilches-Torres A., Lara C.R., Cifuentes G.R., Gómez Castaño J.A. (2022). Use of *Opuntia ficus-indica* Fruit Peel as a Novel Source of Mucilage with Coagulant Physicochemical/Molecular Characteristics. Polymers.

[15] Otálora M.C., Wilches-Torres A., Gómez Castaño J.A. (2023). Mucilage from Yellow Pitahaya (*Selenicereus megalanthus*) Fruit Peel: Extraction, Proximal Analysis, and Molecular Characterization. Molecules.

[16] Gheribi R., Habibi Y., Khwaldia K. (2019). Prickly pear peels as a valuable resource of added-value polysaccharide: Study of structural, functional and film forming properties. Int. J. Biol. Macromol..

[17] Otálora M.C., WilchesTorres A., Gómez Castaño J.A. (2022). Spray-Drying Microencapsulation of Pink Guava (*Psidium guajava*) Carotenoids Using Mucilage from *Opuntia ficus-indica* Cladodes and Aloe Vera Leaves as Encapsulating Materials. Polymers.

[18] Sahoo S., Singh V.K., Uvanesh K., Biswal D., Anis A., Rana U.A., Pal K. (2015). Development of ionic and non-ionic natural gum-based bigels: Prospects for drug delivery application. J. Appl. Polym. Sci..

[19] Carmona J.C., Robert P., Vergara C., Saenz C. (2021). Microparticles of yellow-orange cactus pear pulp (*Opuntia ficus-indica*) with cladode mucilage and maltodextrin as a food coloring in yogurt. LWT Food Sci. Technol..

[20] Garzón G.A., Narváez C.E., Riedl K.M., Schwartz S.J. (2010). Chemical composition, anthocyanins, non-anthocyanin phenolics and antioxidant activity of wild bilberry (*Vaccinium meridionale Swartz*) from Colombia. Food Chem..

[21] Prior R.L., Hoang H., Gu L., Wu X., Bacchiocca M., Howard L. (2003). Assays for hydrophilic and lipophilic antioxidant capacity oxygen radical absorbance capacity (ORAC FL) of plasma and other biological and food samples. J. Agric. Food Chem..

[22] Cunniff P. (1997). Enzymatic-gravimetric method. Official Methods of Analysis of AOAC International.

[23] Meléndez-Martínez A.J., Vicario I.M., Heredia F.J. (2003). Application of tristimulus colorimetry to estimate the carotenoids content in ultrafrozen orange juices. J. Agric. Food Chem..

[24] Jiang M., Zhang Y. (2023). Biopolymer-based encapsulation of anthocyanins as reinforced natural colorants for food applications. J. Sci. Food Agric..

[25] Us-Medina U., Julio L.M., Segura-Campos M.R., Ixtaina V.Y., Tomas M.C. (2018). Development and characterization of spray-dried chia oil microcapsules using by- products from chia as wall material. Powder Technol..

[26] Kaushik V., Roos Y. (2006). Limonene encapsulation in freeze-drying of gum Arabic–sucrose-gelatin systems. LWT Food Sci. Technol..

[27] Tabio-García D., Paraguay-Delgado F., Lardizabal Gutiérrez D., Quintero-Ramos A., Meléndez-Pizarro C.O., Ochoa-Martínez L.A., Sánchez-Madrigal M.A., Ruiz-Gutiérrez M.G., Espinoza-Hicks J.C. (2023). Effectiveness of *Opuntia ficus-indica* mucilage as a carrier agent in microencapsulation of bioactive compounds of Amaranthus hypochondriacus var. Nutrisol. Food Biosci..

[28] Mariod A.A., Mariod A.A. (2018). 20—Gum Arabic Dietary Fiber. Gum Arabic: Structure, Properties, Application and Economics.

[29] Antigo J.L.D., Stafussa A.P., Bergamasco R.D.C., Madrona G.S. (2022). Chia seed mucilage as a potential encapsulating agent of a natural food dye. J. Food Eng..

[30] Tupuna D.S., Paese K., Guterres S.S., Jablonski A., Flôres S.H., Rios A.D.O. (2018). Encapsulation efficiency and thermal stability of norbixin microencapsulated by spray-drying using different combinations of wall materials. Ind. Crop. Prod..

[31] de Araujo Santiago M.C.P., Nogueira R.I., Paim D.R.S.F., Gouvea A.C.M.S., de Oliveira Godoy R.L., Peixoto F.M., Pacheco S., Freitas S.P. (2016). Effects of encapsulating agents on anthocyanin retention in pomegranate powder obtained by the spray drying process. LWT Food Sci. Technol..

[32] Ahmada M., Ashrafa B., Ganib A., Gania A. (2018). Microencapsulation of saffron anthocyanins using β glucan and β cyclodextrin: Microcapsule characterization, release behavior antioxidant potential during *in-vitro* digestion. J. Biol. Macromol..

[33] Machado M.H., Almeida A.D.R., Maciel M.V.D.O.B., Vitorino V.B., Bazzo G.C., da Rosa C.G., Sganzerla W.G., Mendes C., Barreto P.L.M. (2022). Microencapsulation by spray drying of red cabbage anthocyanin-rich extract for the production of a natural food colorant. Biocatal. Agric. Biotechnol..

[34] Bhagya Raj G.V.S., Dash K.K. (2022). Microencapsulation of betacyanin from dragon fruit peel by complex coacervation: Physicochemical characteristics, thermal stability, and release profile of microcapsules. Food Biosci..

[35] Santiago-Adame R., Medina-Torres L., Gallegos-Infante J.A., Calderas F., González- Laredo R.F., Rocha-Guzmán N.E., Ochoa-Martínez L.A., Bernad-Bernad M.J. (2015). Spray drying-microencapsulation of cinnamon infusions (Cinnamomum zeylanicum) with maltodextrin. LWT Food Sci. Technol..

[36] Medina-Torres L., Núñez-Ramírez D.M., Calderas F., González-Laredo R.F., Minjares-Fuentes R., Valadez-García M.A., Bernad-Bernad M.J., Manero O. (2019). Microencapsulation of gallic acid by spray drying with aloe vera mucilage (*Aloe barbadensis* miller) as wall material. Ind. Crop. Prod..

[37] Kwak H.-S., Al Mijan M., Ganesan P. (2014). Application of nanomaterials, nano-and microencapsulation to milk and dairy products. Nano-Microencapsul. Foods.

[38] Burgain J., Gaiani C., Linder M., Scher J. (2011). Encapsulation of probiotic living cells: From laboratory scale to industrial applications. J. Food Eng..

[39] Tonon R.V., Brabet C., Hubinger M.D. (2010). Anthocyanin stability and antioxidant activity of spray-dried açai (*Euterpe oleracea* Mart.) juice produced with different carrier agents. Food Res. Int..

[40] de Campo C., Dick M., dos Santos P.P., Costa T.M.H., Paese K., Guterres S., Rios A.D.O., Flôres S.H. (2018). Zeaxanthin nanoencapsulation with *Opuntia monacantha* mucilage as structuring material: Characterization and stability evaluation under different temperatures. Colloids Surf. A.

[41] Pieczykolan E., Kurek M.A. (2019). Use of guar gum, gum arabic, pectin, beta-glucan and inulin for microencapsulation of anthocyanins from chokeberry. J. Biol. Macromol..

[42] Mahdi A.A., Mohammed J.K., AL-Ansi W., Ghaleb A.D.S., Al-Maqtari Q.-A., Ma M., Ahmed M.I., Wang H. (2020). Microencapsulation of fingered citron extract with gum arabic, modified starch, whey protein, and maltodextrin using spray drying. Int. J. Biol. Macromol..

[43] Soto-Castro D., Gutiérrez Miguel Chávez M., León-Martínez F., Santiago-García P.A., Aragón-Lucero I., Antonio-Antonio F. (2019). Spray drying microencapsulation of betalain rich extracts from *Escontria chiotilla* and *Stenocereus queretaroensis* fruits using cactus mucilage. Food Chem..

[44] Adsare S.R., Annapure U.S. (2021). Microencapsulation of curcumin using coconut milk whey and Gum Arabic. J. Food Eng..

[45] Janiszewska-Turak E., Dellarosa N., Tylewicz U., Laghi L., Romani S., Dalla Rosa M., Witrowa-Rajchert D. (2017). The influence of carrier material on some physical and structural properties of carrot juice microcapsules. Food Chem..

