# Supplementary material for: Spray-Drying Microencapsulation of Andean Blueberry (Vaccinium meridionale Sw.) Anthocyanins Using Prickly Pear (Opuntia ficus indica L.) Peel Mucilage or Gum Arabic: A Comparative Study
Source: Foods. 2023 Apr 27;12(9):1811. doi: 10.3390/foods12091811 (PMC10178270; doi:10.3390/foods12091811)
Supplement: Supplementary file 1 [file foods-12-01811-s001.zip › foods-2329690-supplementary.pdf]

foods

MDPI

Article

Supplementary Materials

Spray-drying Microencapsulation of Andean Blueberry (*Vaccinium meridionale* Sw.) Anthocyanins using Prickly Pear (*Opuntia ficus indica* L.) Peel Mucilage or Gum Arabic: a Comparative Study

Maria Carolina Otálora [1,*], Andrea Wilches-Torres [1] and Jovanny A. Gómez Castaño [2,*]

[1] Grupo de Investigación en Ciencias Básicas (NÚCLEO), Facultad de Ciencias e Ingeniería, Universidad de Boyacá, Tunja 150003, Colombia
[2] Grupo Química-Física Molecular y Modelamiento Computacional (QUIMOL®), Escuela de Ciencias Químicas, Universidad Pedagógica y Tecnológica de Colombia, Tunja 150003, Colombia; grupo.quimol@uptc.edu.co
* Correspondence: marotalora@uniboyaca.edu.co (M.C.O.); jovanny.gomez@uptc.edu.co (J.A.G.C.)

Citation: To be added by editorial staff during production.

Academic Editor: Firstname Lastname

Received: date
Revised: date
Accepted: date
Published: date

Copyright: © 2023 by the authors. Submitted for possible open access publication under the terms and conditions of the Creative Commons Attribution (CC BY) license (https://creativecommons.org/licenses/by/4.0/).

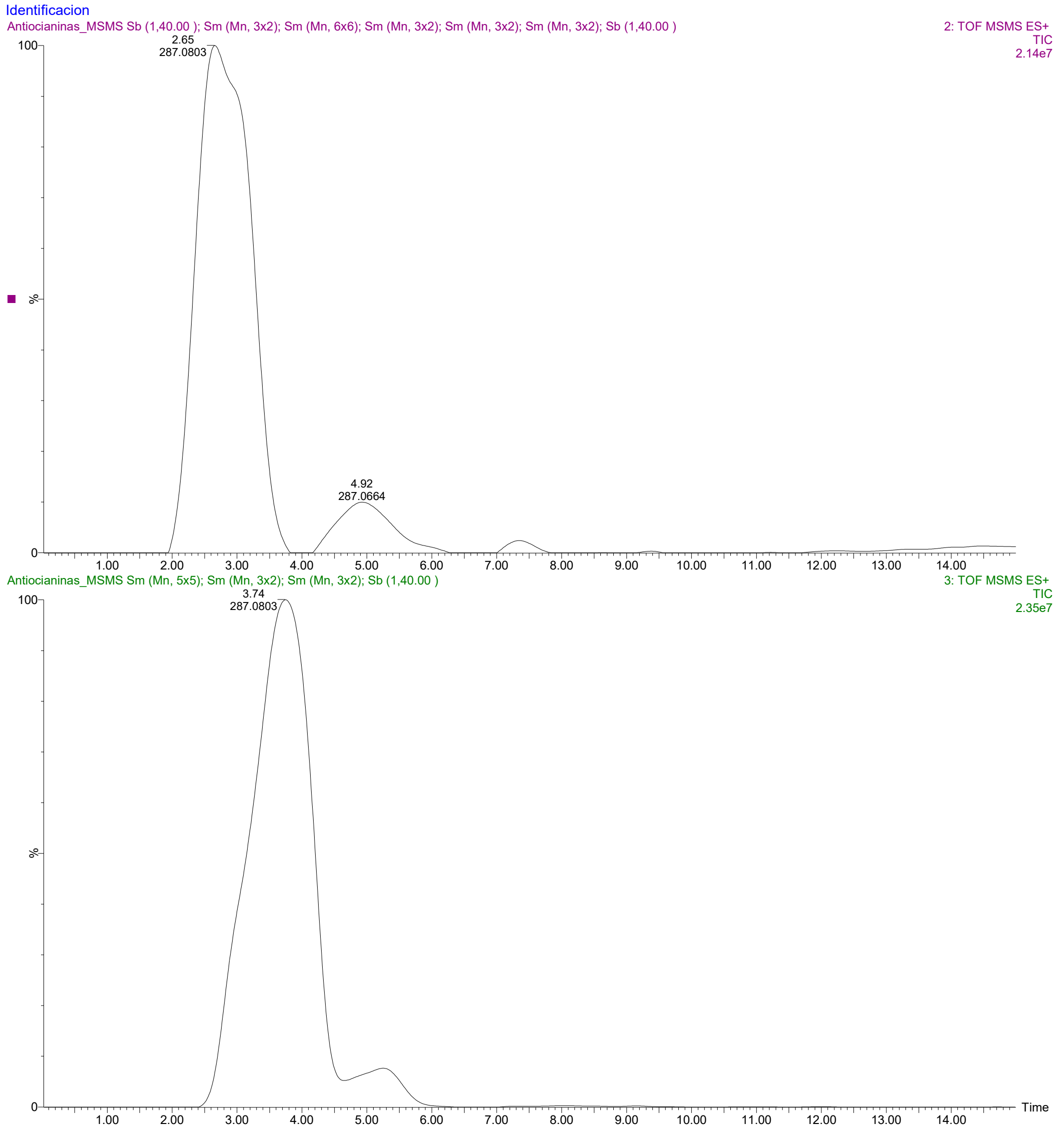

Figure S1. HPLC chromatograms of the two main anthocyanins (i.e., cyanidin-3-O-alpha-arabinopyranoside ($C_{20}H_{19}O_{10}$) (**top**) and cyanidin-3-O-galactoside ($C_{21}H_{21}O_{11}$) (**bottom**)) identified in the lyophilized pulp of Colombian Andean blueberry (*Vaccinium meridionale Sw*).

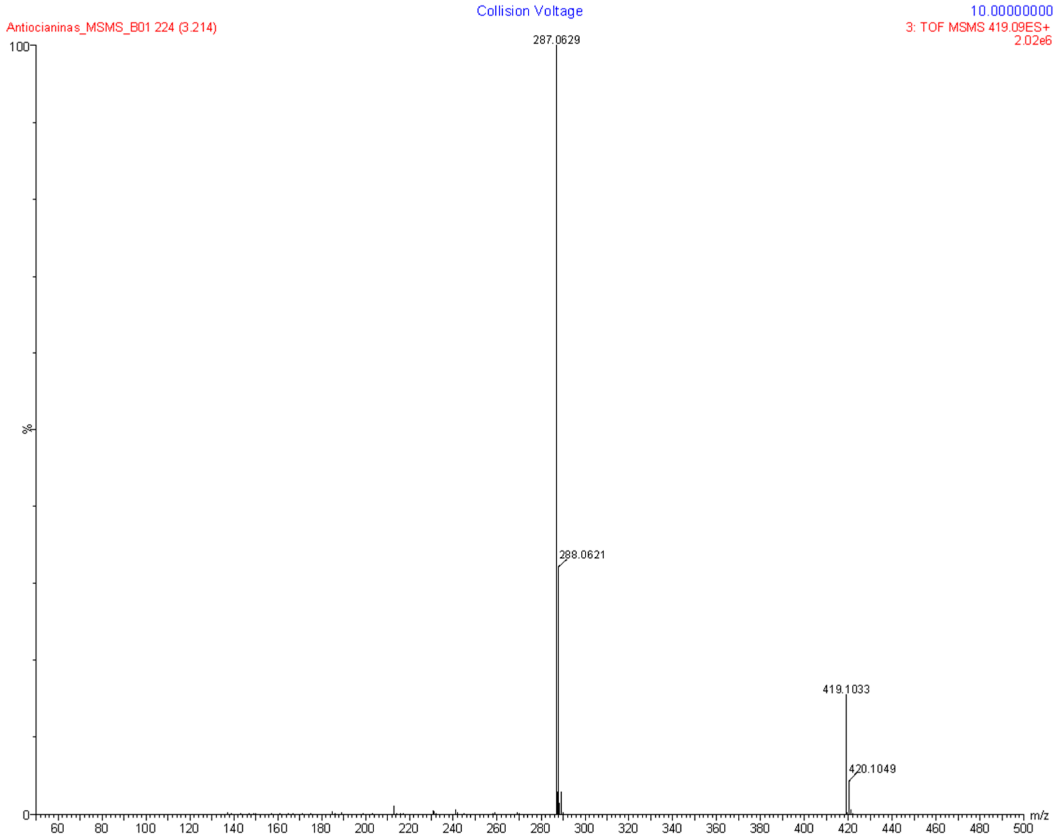

Figure S2. Mass spectrum of the anthocyanin cyanidin-3-O-alpha-arabinopyranoside ($C_{20}H_{19}O_{10}$) identified in the pulp of Colombian Andean blueberry (*Vaccinium meridionale Sw*) determined using the HPLC/MS-MS technique.

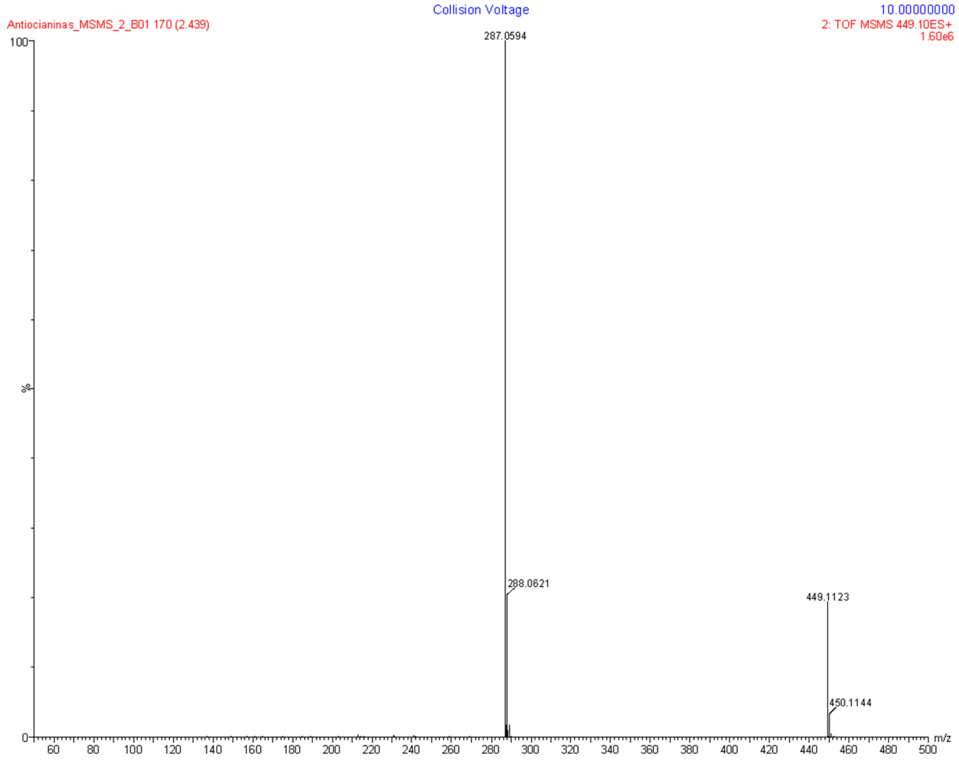

Figure S3. Mass spectrum of the anthocyanin cyanidin-3-O-galactoside ($C_{21}H_{21}O_{11}$) identified in the pulp of Colombian Andean blueberry (*Vaccinium meridionale Sw*) determined using the HPLC/MS-MS technique.

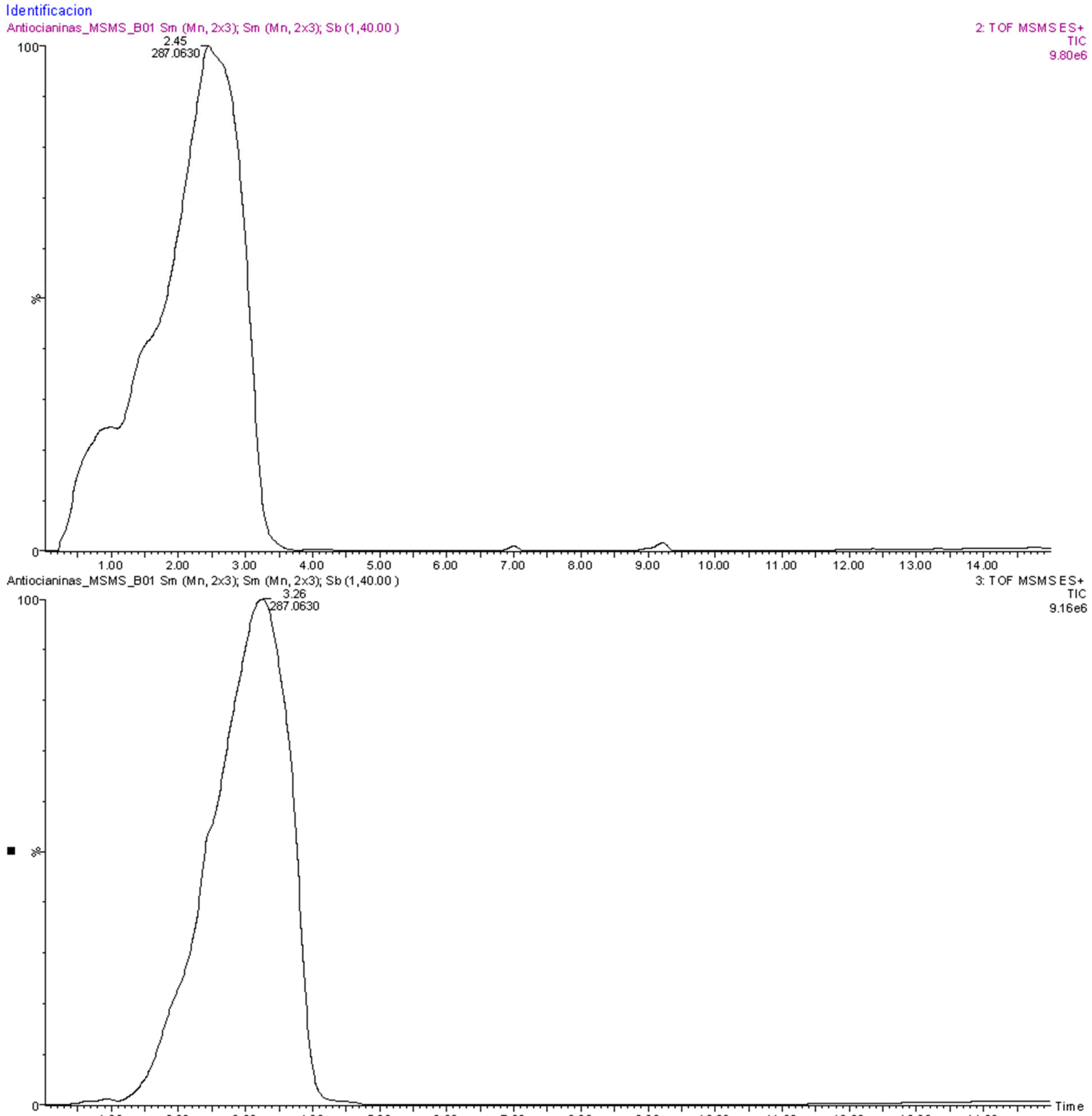

Figure S4. HPLC chromatograms of the two main anthocyanins (i.e., cyanidin-3-O-alpha-arabinopyranoside ($C_{20}H_{19}O_{10}$) (**top**) and cyanidin-3-O-galactoside ($C_{21}H_{21}O_{11}$) (**bottom**)) identified in the Andean blueberry (*Vaccinium meridionale Sw*) microcapsules using OFI mucilage as encapsulated material.

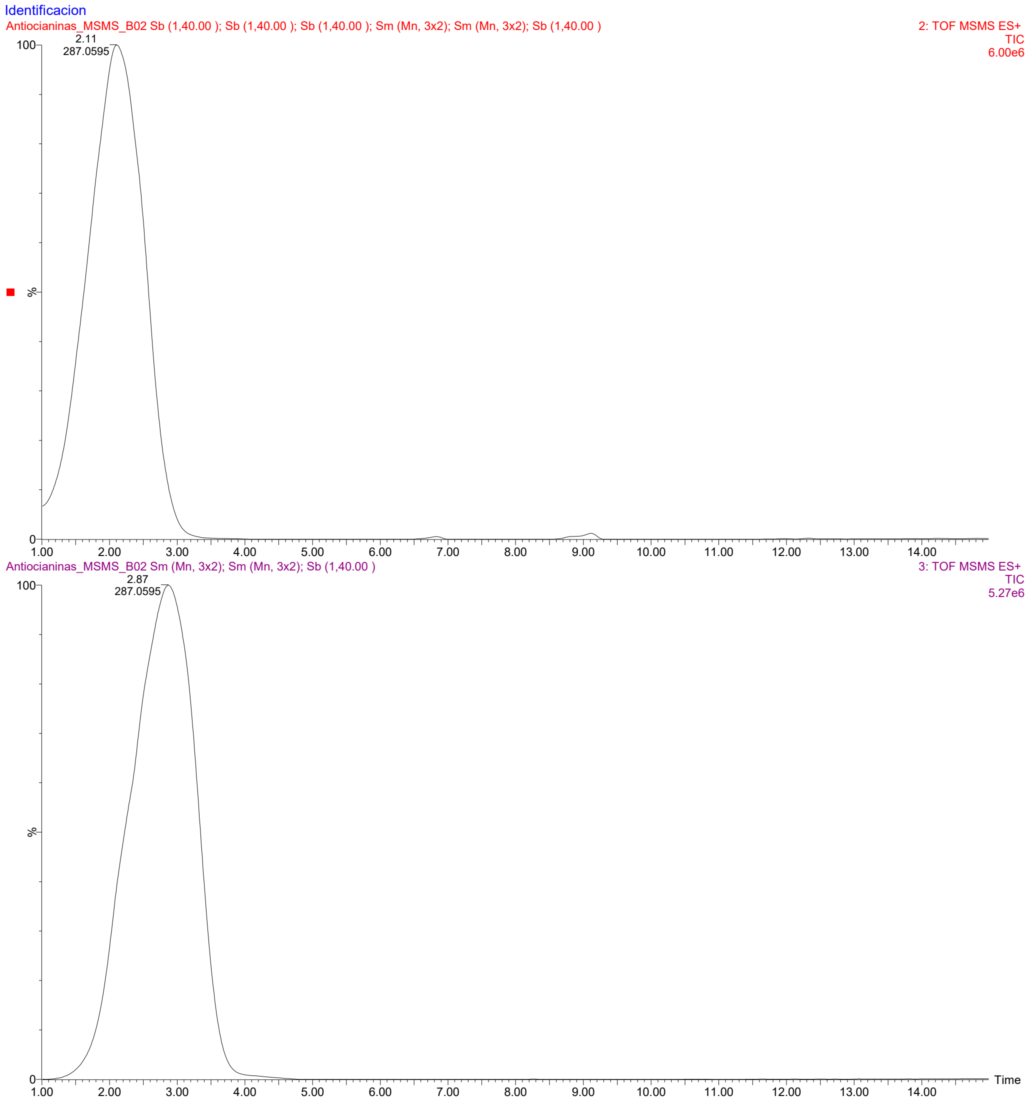

Figure S5. HPLC chromatograms of the two main anthocyanins (i.e., cyanidin-3-O-alpha-arabinopyranoside ($C_{20}H_{19}O_{10}$) (**top**) and cyanidin-3-O-galactoside ($C_{21}H_{21}O_{11}$) (**bottom**)) identified in the Andean blueberry (*Vaccinium meridionale Sw*) microcapsules using gum arabic as encapsulated material.
